# How defense rhetoric escalates intergroup conflict

**DOI:** 10.1016/j.isci.2025.113749

**Published:** 2025-11-05

**Authors:** Luuk L. Snijder, Jörg Gross, Carsten K.W. De Dreu

**Affiliations:** 1 Faculty of Behavioural and Social Sciences, University of Groningen, Groningen, the Netherlands; 2 Social and Economic Psychology, University of Zurich, Zurich, Switzerland; 3 Faculty of Economics and Business, University of Groningen, Groningen, the Netherlands; 4 Behavioural Ecology and Sociobiology Unit, Leibniz Institute for Primate Research, Göttingen, Germany

**Keywords:** Social sciences, Decision science, Political science, Research methodology social sciences, Sociology

## Abstract

The first victim of conflict is often the truth. Using 261 manifestos from real-world conflicts, we show that leaders frequently misrepresent strategic intentions in their calls to arms and that such misrepresentation is asymmetric. Leaders of attacking parties hide their primary objective for exploitation behind claims of self-defense. Experiments show that self-defense rhetoric, true and false, is readily believed by naive participants (*n* = 252 and 312; pre-registered) and large language models (2,162 resamples), and boosts support for leaders’ stated cause. Leaders in intergroup contests frequently invoke the need for self-defense even when outgroups pose no actual threat. This leads followers to increase their costly contributions to fight non-threatening outgroups, making their (deceptive) leaders prevail in increasingly intense and wasteful intergroup conflicts. Our findings elucidate when leaders resort to false signaling, why they do so, and how it can escalate conflicts that benefit warring leaders at significant cost to society.

## Highlights

• Leaders often justify aggression with false claims of self-defense
• The general public, and LLMs, easily fall for false defense rhetoric
• Framing attack as defense makes followers fight harder and win more often
• False signaling benefits attacking leaders while harming everyone else

## Graphical abstract

**Figure:**
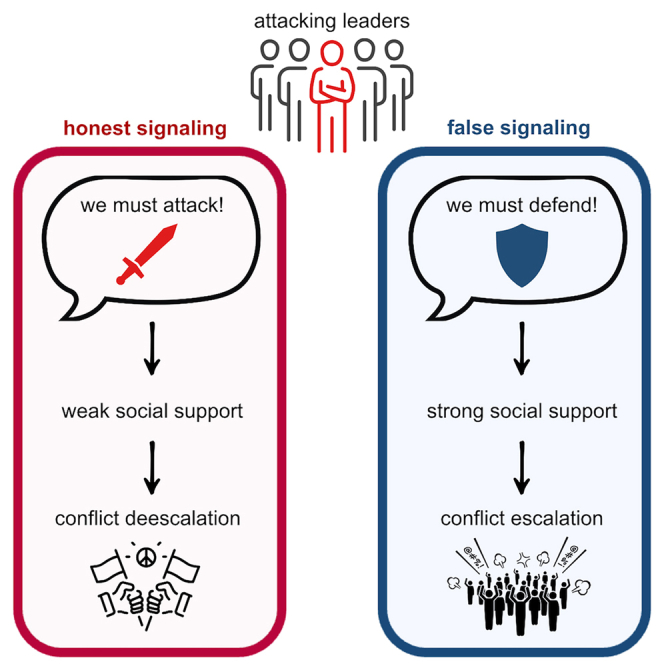

## Introduction

Intergroup conflict and warfare are a persistent and tragic thread of human history.^1^^,^^2^ Globally, and since 2010 alone, more than four million lives have been lost in direct acts of warfare (see Methods S1^3^) and the socio-economic impacts of conflict are difficult to overestimate.^4^^,^^5^^,^^6^ In sharp contrast to the frequently celebrated human capacities for empathy and cooperation,^7^^,^^8^^,^^9^ the prevalence and wastefulness of intergroup conflict poses a profound puzzle for psychologists: why, and under what circumstances, do humans unite in groups to inflict harm on others?

When faced with enemy threats, the answer appears straightforward. Defending oneself and one’s group against outside threats is *jus ad bellum*—morally, legally, and psychologically permissible.^10^^,^^11^ Furthermore, when faced with an external threat, the interests of the individual and the group are often aligned, creating a shared fate and purpose that can strongly motivate and unite leaders and group members to fight for themselves and their group.^12^^,^^13^^,^^14^^,^^15^

The challenge, rather, is to understand how leaders manage to mobilize support for aggression to seize territory, resources, or political power from otherwise non-threatening groups. Such actions may benefit leaders directly, by gaining power, status, or wealth.^16^^,^^17^ However, unlike defensive action, initiating otherwise unprovoked aggression toward outsiders may be difficult to justify on moral grounds.^18^ And while participating in conflict can be personally costly and risky, participating in unprovoked aggression typically offers ordinary group members few, if any, direct benefits compared to defending against outside threat. Therefore, and unlike coalitionary defense, coalitionary attacks come with a range of coordination and free-rider problems, as well as psychological resistance that individuals and their leaders would need to overcome.^6^^,^^16^^,^^17^^,^^19^^,^^20^^,^^21^^,^^22^^,^^23^^,^^24^

To overcome these difficulties, and to unite individuals for coalitionary attacks on outgroups, leaders may motivate conflict participation by offering stronger extrinsic incentives (e.g., status, wealth, or ideological rewards). In addition, or alternatively, leaders aiming to conquer and exploit outgroups may disguise their true objectives and falsely invoke the need for self-defense when issuing calls to arms. If convincing enough, leaders would change the psychology of group members such that aggressive actions toward outsiders are seen as protective of the individual’s personal interests and, therefore, needed, justifiable, intrinsically motivating, and morally permissible.

While resonating with common intuition that leaders sometimes “spin” war narratives and resort to defense rhetoric, we lack a systematic analysis of war rhetoric and its effects on follower psychology and behavioral decision-making. Our current aims, accordingly, are to gain better insight into (1) whether and how often revisionist attackers falsely invoke defense rhetoric; (2) whether individuals can detect and resist such deceptive framing or whether they tend to “buy into” these misleading narratives, and (3) how deceptive rhetoric impacts actual conflict dynamics. We pursued these aims with, first, archival analyses of historical war manifestos (1508–1941) that allowed us to quantify how often leaders justify their resort to war with claims of self-defense, even when their true motive is attack. Second, we investigate whether naive individuals can accurately discern deceptive rhetoric or not. For this purpose, participants are presented with decontextualized excerpts from historical speeches delivered at the onset of wars. They are asked to identify each excerpt as originating from an attacker or a defender and to indicate their support for the respective cause. Finally, we performed an interactive, incentivized attacker-defender contest in which leaders could misrepresent their group’s position in the conflict. This enables us to test whether leaders falsely signal self-defense, and how such deception causally impacts followers’ contributions to intergroup conflict. Findings combined reveal when leaders are more likely to engage in deceptive rhetoric and false signaling, and how coalitionary aggression on non-threatening outgroups can emerge and persist.

## Results

### Archival analysis

**Figure 1:**
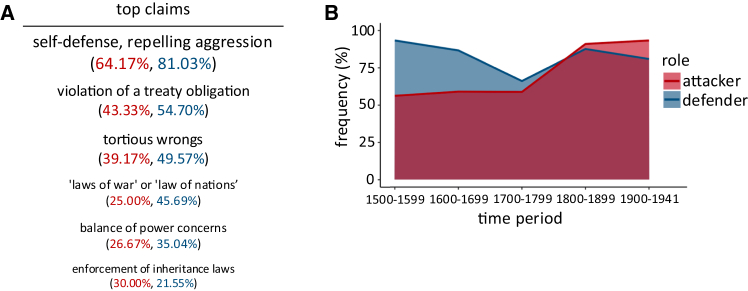
Leaders use defensive rhetoric even when they aim to conquer and exploit (A) Most frequently used claims in war manifestos by both attackers (red) and defenders (blue). The three most frequently used reasons to resort to war were the same for attackers and defenders. (B) The usage of “self-defense and repelling aggression” claims in war manifestos by attackers (red) and defenders (blue) is consistently high across the time-period analyzed.

In a first step, we coded for war manifestos issued between 1508 and 1941^25^^,^^26^ whether the issuing leader’s most likely true reason for staging war was either revisionist attack or non-revisionist defense (see STAR Methods). Text analyses of war manifestos revealed consistent references to enemy threats and the need for defense and protection. Importantly, this was also the case when issuing countries’ actual objectives were conquest, subjugation, and exploitation (Figure 1). In fact, both state leaders facing enemy hostilities (non-revisionist defenders; *n* = 117) and those seeking to aggressively exploit non-threatening neighbors (revisionist attackers; *n* = 120) referenced the need for self-defense in 81.03% and 64.17% of the cases, respectively (Figure 1A). Both frequencies exceeded 50%, underscoring the widespread use of self-defensive claims by both attackers and defenders (binomial tests; non-revisionist defenders, *p* < 0.001, revisionist attackers, *p* = 0.002; Table S1). Of note is also a marked discontinuity around 1700 for attackers (Figure 1B) after which references to “self-defense and repelling aggression” sharply increased. This increase in falsely signaling closely follows revisions of international law and state sovereignty introduced in the Peace of Westphalia of 1648 (see discussion and Table S2).

**Figure 2:**
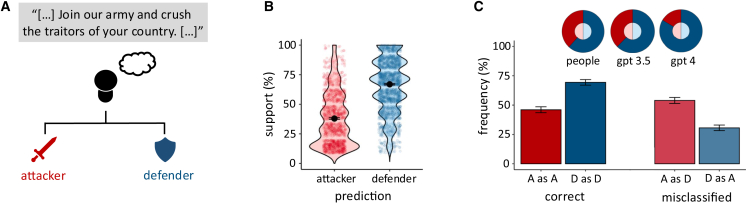
Human participants and large language models are deceived by rhetoric that falsely claims enemy threat and need for defense (A) Participants (*n* = 252) classified excerpts from public speeches at the onset of war (ranging from 50 to 139 words; see Methods S2 for the excerpts used) as either revisionist or non-revisionist and indicated their confidence in their classification and support for each cause. (B) Violin plot with the support for leaders’ causes based on whether participants perceived them to be revisionist attackers (red) or non-revisionist defenders (blue). Participants reported stronger support for leaders’ causes when they perceived them as defenders rather than attackers. Dots represent individual data points showing the relative support for each excerpt based on the perceived position. Error bars indicate the 95% CI. (C) Classification of attacker (red) and defender (blue) speeches. Participants correctly classified most defender speeches (69.38%) but misclassified attacker speeches (53.97%) more often than that they classified them correctly (46.03%). Pie charts illustrate how often participants (attack = 38.33%, defense = 61.67%), ChatGPT-3.5 (attack = 37.64%, defense = 62.36%), and ChatGPT-4 (attack = 16.53%, defense = 83.47%) classified speech excerpts as belonging to an attacking (dark red) or defending (dark blue) leader, despite an equal distribution of speeches from attackers and defenders (50%). Error bars indicate the standard error of the mean.

Whereas our archival analysis revealed that leaders, throughout history, frequently and untruthfully invoke enemy threat and the need for self-defense, the important question is whether such false signaling is convincing and successful in garnering support. If it is, we should see stronger support for leaders who portray their war as needed for self-defense independent of whether there is an actual enemy threat or not. We tested these possibilities in experiment 1 with both human participants and large language models (Figure 2A).

### Experiment 1

As predicted, participants reported stronger support for leaders’ causes when they perceived them to be non-revisionist defenders (Figure 2B; multilevel model [MLM], *b* = 1.92, *p* < 0.001, 95% confidence interval [CI]: [1.82, 2.01]; Table S3). Crucially, this was independent of the leader’s actual revisionist or non-revisionist stance in the conflict (MLM, *b* = 0.05, *p* = 0.314, 95% CI: [−0.04, 0.13]; Table S3). These findings suggest that the perception of being a defender, rather than the objective position, plays a critical role in shaping support for war.

Even though we presented an equal number of speeches from revisionist attackers and non-revisionist defenders, participants classified more speech excerpts as belonging to a non-revisionist defender (61.67%) than revisionist attacker (38.33%) (Figure 2C). Participants accurately identified 69.38% of non-revisionist defenders (binomial, MLM, *b* = 0.85, *p* < 0.001, 95% CI: [0.73, 0.98]; Table S4), but only identified 46.03% of revisionist attackers correctly (binomial MLM, *b* = −0.17, *p* = 0.005, 95% CI: [−0.28, −0.05]; Table S4). This misclassification is unlikely to result from inattention or disinterest, as participants were incentivized for accuracy. Instead, this systematic deviation suggests a strong bias in perception: participants were more likely to classify leaders as non-revisionist defenders, even when they were, in fact, revisionist attackers. And as shown, perceiving leaders to be non-revisionist defenders, even when in reality they are revisionist attackers, increased support for their causes.

Results replicated when we replaced human participants with large language models pre-trained on knowledge sources such as Wikipedia, news articles, books, or scientific publications (ChatGPT-3.5 and -4). Like human participants, chatbots reported to “trust” leaders they classified as non-revisionist defenders more than those classified as revisionist attackers (regression; GPT-3.5: *b* = 1.10, *p* < 0.001, 95% CI: [1.01, 1.19]; GPT-4: *b* = 1.01, *p* < 0.001, 95% CI: [0.81, 1.22]; Tables S5 and S6). And like humans, chatbots misclassified excerpts from revisionist speeches as coming from non-revisionist defenders—while 50% of the presented excerpts were from a non-revisionist defender, 62.36% (GPT-3.5) and 83.47% (GPT-4) of war excerpts were classified as non-revisionist. Finally, we again observed above chance likelihood of correctly attributing excerpts to non-revisionist defenders (GPT-3.5: 68.33%; binomial test, *p* < 0.001, GPT-4: 88.89%; binomial test, *p* < 0.001), and below chance likelihood of correctly attributing excerpts to revisionist attackers (Figure 2C; GPT-3.5: 43.61%, binomial test, *p* = 0.001; GPT-4: 21.94%; binomial test, *p* < 0.001; Tables S7 and S8). In short, both human participants and chatbots trained on large language models were deceived by self-defense rhetoric when the true motive behind the leaders’ decision to wage war was revisionist. Moreover, both human participants and chatbots reported higher (moral) support for those leaders who more prominently invoked enemy threat and the need for self-defense.

Experiment 1 shows that leaders falsely signal conflict as needed for self-defense, and that naive participants are not only deceived by such false rhetoric but also increase their support for the leader’s stated cause. What experiment 1 cannot reveal, however, is whether being in a revisionist attacker position causally influences tendencies in leaders to false signaling and whether and how such false signaling shapes the dynamics and costs of intergroup conflict. If that is the case, it should also follow that the opportunity of false signaling and deceptive rhetoric intensifies intergroup conflict, increases its waste and the likelihood that outgroups are defeated and exploited. To test these (pre-registered) hypotheses, we performed a laboratory experiment with an interactive and fully incentivized attacker-defender contest.^6^

### Experiment 2

**Figure 3:**
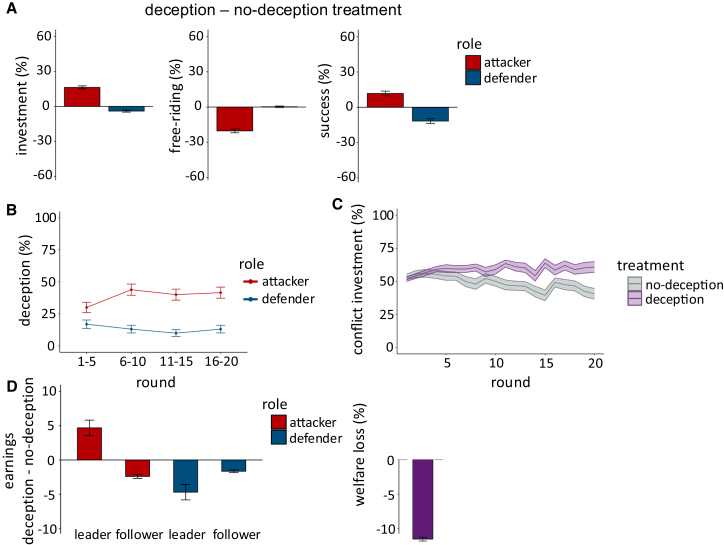
Falsely signaling self-defense escalates intergroup conflict (A) Difference in conflict investments, free-riding (i.e., investing zero resources in conflict), and winning probabilities between the deception and no-deception treatments for attackers (red) and defenders (blue). (B) Relative frequency of attacking (red) and defending (blue) leaders deceiving their followers in the deception treatment across rounds. In the first five rounds, leaders of attacking groups already deceived their followers in 30% of rounds which increased to 41.54% in the last five rounds. (C) Followers’ contributions to conflict over time in the deception (purple) and no-deception (gray) treatment. In the no-deception treatment, followers’ conflict contributions decreased over time. In contrast, conflict contributions, and hence, waste of conflict, increased over time in the deception treatment. (D) Difference in participants’ average earnings between the deception and no-deception treatments for attackers (red) and defenders (blue). Only leaders of attacking groups benefited from the possibility of deception. All other parties earned less, resulting in an overall welfare loss of 11.06% (purple). Error bars and bands indicate the standard error of the mean.

When leaders had to communicate their position truthfully, i.e., when false signaling was not possible, conflict evolved in line with earlier results.^6^^,^^19^^,^^20^^,^^21^^,^^22^^,^^27^ Individuals in attacker groups contributed, on average, less than those in defender groups (Figure 3A; MLM, *b* = −3.84, *p* < 0.001, 95% CI: [−4.23, −3.44]; Table S9), and more often contributed nothing to conflict (viz. free-riding; multilevel logistic model [MLLM], *b* = 4.33, *p* < 0.001, 95% CI: [3.72, 5.04]; Table S10). Attack was less coordinated and less forceful compared to defense, as defenders had a stronger common interest in defending their resources that was lacking for attackers. As a result, attacker groups were less successful than defenders. In 75.38% of the rounds, defenders successfully prevented attackers from taking their resources (MLLM, *b* = −2.24, *p* < 0.001, 95% CI: [−2.52, −1.96]; Table S11). Consequently, attacking leaders earned less than defending leaders (MLM, *b* = −20.31, *p* < 0.001, 95% CI: [−22.35, −18.26]; Table S12). In sum, when attacker groups were aware that their goal in conflict was to appropriate the resources of another (non-hostile) group, their attempts were uncoordinated and often unsuccessful. Over rounds, conflict expenditures also decreased (MLM, *b* = −0.15, *p* < 0.001, 95% CI: [−0.18, −0.11]; Table S13), providing evidence for the conjecture that intergroup conflict should be rather rare when one group is openly attacking another on no moral grounds.

Leaders in the deception treatment, on the other hand, could try to manipulate what their followers believed their position in the contest was. Here, and in line with our archival analyses of war manifestos, leaders disproportionately often invoked ingroup defense as the position their group was in. While defending leaders rarely misled their followers (13.27%), attacking leaders falsely portrayed their group’s position as defense in 38.85% of the contest rounds (Figure 3B; MLM, *b* = 1.75, *p* < 0.001, 95% CI: [1.40, 2.11]; Table S14). This false signaling seemed to be intentional. When leaders in an attacking position reported their group’s position as defense, they expected their followers to contribute more to conflict (MLM, *b* = 1.89, *p* < 0.001, 95% CI: [1.19, 2.58]; Table S15). More crucially, false signaling worked. When attacking leaders misleadingly invoked self-defense, their followers indeed increased their contributions to the conflict (MLM, *b* = 3.57, *p* < 0.001, 95% CI: [2.96, 4.17]; Table S16), free-rode less (MLM, *b* = −3.03, *p* < 0.001, 95% CI: [−4.86, −2.10]; Table S17), and were more likely to win the contest (MLLM, *b* = 0.88, *p* < 0.001, 95% CI: [0.55, 1.22]; Table S18) (Figure 3A), also in comparison to the no-deception treatment (MLLM, *b* = 0.57, *p* < 0.001, 95% CI: [0.25, 0.91]; Table S19).

False signaling escalated intergroup conflict and resulted in an overall loss of 11.06 percentage points in social welfare compared to the no-deception treatment. While conflict contributions declined over time in the no-deception treatment (MLM, *b* = −0.15, *p* < 0.001, 95% CI: [−0.18, −0.11]; Table S13), they increased in the deception treatment (Figure 3C; MLM, *b* = 0.20, *p* < 0.001, 95% CI: [0.15, 0.25]; Table S13). Individuals in attacker groups in the deception treatment thus wasted more of their resources on conflict and earned less especially on rounds when their leader misled them about their position in the conflict (MLM, *b* = −1.88, *p* < 0.001, 95% CI: [−2.67, −1.09]; Table S20). Ironically, the opposite was the case for their leaders, who earned more when they were permitted to deceive (compared to no-deception: MLM, *b* = 4.69, *p* = 0.002, 95% CI: [1.75, 7.64]; Table S21), and especially when they made use of deception (Figure 3D; MLM, *b* = 6.44, *p* < 0.001, 95% CI: [2.87, 9.95]; Table S22).

## Discussion

When declaring war and issuing calls to arms, leaders often manipulate, distort, or even fabricate reasons to justify violent aggression against other nations or groups. For instance, U.S. President George W. Bush publicly justified the invasion of Iraq by suggesting the presence of weapons of mass destruction—a claim his administration likely knew to be false.^28^ More recently, Russian President Vladimir Putin framed his “special military operations” in Ukraine as a defense against Neo-Nazis in the Ukrainian government allegedly persecuting Russian minorities—an unfounded assertion that Putin himself may not have believed.^29^ War rhetoric can be selectively one-sided and, at times, delusional.

Falsehoods and self-defense rhetoric are not unique to contemporary world leaders. They have been employed throughout history and as shown here, serve two key purposes. Strategically, when group members believe they are defending themselves, they have a stronger aligned interest to prevail against the enemy. Their personal outcomes are tied to the success of the group that, as observed in our experiment, diminishes free-riding and helps coordination. Psychologically, invoking enemy threat and the need for defense, as shown, can increase support and make causing others harm morally permissible, leveraging the stronger motivation to avoid loss than to acquire otherwise equivalent gain.^16^^,^^17^^,^^19^^,^^30^^,^^31^^,^^32^^,^^33^^,^^34^

That falsely invoking self-defense targets the immorality and illegality of attacking non-threatening outgroups fits with archival analyses. We observed a marked increase in the tendency to (falsely) use self-defense and repelling aggression as justifications to initiate war around 1700 (Figure 1B; logistic regression, *b* = 0.005, *p* = 0.013, 95% CI: [0.001, 0.01]; Table S2). This increase closely follows the Peace of Westphalia of 1648. The treaty ended the thirty years war in Central Europe with the explicit recognition that it is legally and morally prohibited to aggressively invade other states.^35^ A similar pattern may have emerged after the adoption of the UN Charter in 1945, which also prohibited attacks.^11^ In response to these institutional changes, revisionist attackers may have increasingly resorted to dishonestly framing their actions as self-defense.

A remaining question is why people act on their leaders’ deceptive rhetoric. One straightforward explanation is that, all else being equal, individuals’ risk to lose more by mistakenly dismissing a leader’s call to defend and protect the group. Such tendencies of “erring on the safe side” may also lead individuals into motivated reasoning, believing and supporting the leader to justify their own actions. We could find some evidence for this possibility in our laboratory experiment. After the contest, followers could donate points to their leader as a form of (costly) support (STAR Methods). Donations in the deception treatment were independent of the number of rounds that their leader honestly revealed the group’s position in the contest (MLM, *b* = −0.05, *p* = 0.444, 95% CI: [−0.19, 0.08]; Table S23). Furthermore, followers overestimated the number of rounds that their group had been in a defensive position (*M* = 11.54; one-sample *t*(103) = 5.21, *p* < 0.001). As donations were personally costly, and correct estimates yielded extra payment, these results suggest that followers were oblivious to their leader’s integrity and underestimated their leaders’ use of deception.

Whereas motivated erring on the safe side may explain participant behavior in intergroup contest experiments, it cannot fully explain the “self-defense bias” we observed when uninvolved human participants and large language models classified excerpts from war speeches. One possibility is that for both humans and large language models cues of threat and risk are more “attention-grabbing” than cues of opportunities and safety.^30^^,^^31^^,^^32^^,^^33^ If true, falsely invoking enemy threat and the need for self-defense can be deeply effective, and perpetuate, because it harps on fundamental “biases” in cognition and behavior. Threat-sensitivity can have survival functionality,^33^ yet, as shown here, it can also lure individuals into fighting conflicts that benefit leaders and destroy social welfare.

Like human participants, GPT-3.5 and GPT-4 misidentified attacker rhetoric as defensive and expressed more trust in leaders who invoked the need for defense. As the mass adoption of these models for information gathering increases, this result highlights the importance for “naive” users to approach outputs from large language models critically, especially when engaging with politically sensitive content.

### Limitations of the study

To identify which side was the attacker in our archival analysis, we coded the first state to initiate military force as the attacker. This approach (1) aligns with definitions of asymmetric conflict and warfare in political science and international relations,^6^^,^^36^ (2) avoids subjective judgments about leaders’ intentions, and (3) enables consistent classification across a large set of manifestos. However, it also oversimplifies the more complex psychological and political factors behind conflict initiation. First, leaders may sincerely perceive their actions as defensive, even when they are not. Such defensive self-deception may even have functional value by enhancing leaders’ ability to persuade others and enable them to communicate with greater conviction.^37^^,^^38^^,^^39^ Second, being classified as *revisionist* in datasets such as the militarized interstate disputes (MID) does not inherently imply aggressive intent.^17^^,^^40^ Rather, it reflects an effort to change the status quo, which in some conflicts may apply to both parties (e.g., during disputes where territories hold symbolic or historical meaning^41^^,^^42^). Third, many conflicts unfold over extended periods of time and involve repeated cycles of provocation, retaliation, and negotiation, making it difficult to assign fixed labels of attacker and defender.

While these limitations cannot be fully addressed through our archival analysis alone, our experimental design provides a controlled test of strategic deception: leaders were randomly assigned attacker or defender roles and knew their group’s actual position. Even under these conditions, where self-deception is very unlikely, 38.9% of attacking leaders falsely signaled their position as defense. This finding offers robust evidence that defense rhetoric is not merely a result of misperception or self-deception, but also a strategic choice. Therefore, Study 2 likely offers a conservative estimate of the use of deception: in real-world settings, where self-deception may occur alongside intentional misrepresentation, the use of misleading narratives could be even more widespread. Further investigation is needed to identify when and how leaders come to believe their own justifications for war, and how self-deception, compared to intentional deception, affects the mobilization of followers and the persistence of conflict.

In our experiment, leaders were appointed, and re-election was not part of the experimental design. Furthermore, our set-up made it difficult if not impossible for followers to “find out” that their leader was honest, or deceptive. Yet when deception can be detected, and being detected can damage reputations and opportunities (e.g., for being re-elected as leader^43^), leaders may become less inclined to deceive their followers and more likely to honestly convey their group’s position in the conflict. Future research could examine whether leader rhetoric becomes less deceptive when followers are likely to detect deception, and detecting deception adversely impacts leaders.

While our stylized context allows the manipulation of leaders’ ability to deceive, thereby isolating its causal effect on conflict participation and escalation, generalizing these findings to real-world conflicts warrants caution. In our experiment, followers learned about their group’s position (attacker or defender) solely through their leader. This mimics autocratic regimes with full control of information provision or the early stages of conflict where access to alternative information is limited and individuals must rely on leadership cues. In other situations, however, individuals may revise their beliefs based on independent media, opposition voices, or battlefield developments. The US invasion of Iraq in 2003 is illustrative: while initial public support for the invasion was strongly based on claims about weapons of mass destruction, it declined sharply when independent media reports proved these claims to be false.^28^ Notably, deceptive leaders may attempt to discredit or suppress such information to prevent backlash. Future research could investigate how individuals revise or abandon beliefs as new information emerges, and how misinformation can be effectively corrected.

Whereas our analysis focused on leaders and their followers in general, followers often have different stakes in intergroup conflict, and these may affect the psychological and strategic impacts of defense rhetoric. For example, individuals directly involved in the conflict, such as soldiers or civilians in high-risk areas, may act on leaders’ cues out of strategic precaution—even if they suspect deception—because they could lose more by mistakenly dismissing a leader’s call to defend and protect the group. Over time, their participation may reinforce belief in the narrative through motivated reasoning. In contrast, more distant stakeholders, such as citizens in safer regions or third-party observers, may be less susceptible to these pressures and more willing to question official claims, particularly when alternative information is available. Future research could examine how physical and psychological proximity influence susceptibility to war rhetoric.

### Conclusion

To conclude, findings explain why deception, propaganda, and fake news^44^^,^^45^^,^^46^ are pervasive in intergroup conflicts, and give a mechanistic explanation of how portrayal of victimhood can escalate conflict. When both sides see themselves as righteous defenders, this perception is not only psychologically self-serving but also generates ambiguities that leaders can exploit, often at a significant cost to society as a whole.

## Resource availability

### Lead contact

Requests for further information and resources should be directed to and will be fulfilled by the lead contact, Luuk L. Snijder (l.l.snijder@rug.nl).

### Materials availability

All study materials are publicly available (https://osf.io/DE34J/).

### Data and code availability

• All de-identified data from the archival analysis, experiment 1, and experiment 2 are publicly available (https://osf.io/DE34J/).
• All analysis scripts for the archival analysis, experiment 1, and experiment 2 are publicly available (https://osf.io/DE34J/).
• Codebooks, experimental instructions, survey materials, and war speech excerpts are publicly available (https://osf.io/DE34J/).

## Acknowledgments

The authors would like to thank Anna Wickenkamp for her help with coding and developing materials, and Niels van Doesum and Gisela Hirschmann for feedback on the study design and interpretation. This project has received funding from the Netherlands Science Foundation (10.13039/501100003246NWO SPI-57-242) to C.K.W.D.D., and the 10.13039/501100000781European Research Council (ERC) under the European Union’s Horizon 2020 research and innovation program to C.K.W.D.D. (AdG agreement no. 785635) and to J.G. (StG agreement, 10.13039/501100007352SBFI no. MB23.0003).

## Author contributions

All authors contributed equally.

## Declaration of interests

The authors declare no competing interests.

## STAR★Methods

### Key resources table

**Table:** 

REAGENT or RESOURCE	SOURCE	IDENTIFIER
**Deposited data**		

War Manifestos Database (1508–1941)	Hathaway et al.^25^	https://documents.law.yale.edu/manifestos
War manifestos codebook & analysis scripts	This paper (OSF)	https://osf.io/DE34J/
Militarized Interstate Disputes Dataset (MID5, 1816–2014)	Correlates of War Project^36^	https://correlatesofwar.org/data-sets/MIDs
Justifications of War Archive	TeachWar^47^	https://teachwar.wordpress.com/resources/war-justifications-archive/
Experiment 1 experiment code, data, and analysis scripts	This paper (OSF)	https://osf.io/DE34J/
Experiment 2 experiment code, data, and analysis scripts	This paper (OSF)	https://osf.io/DE34J/

**Software and algorithms**		

oTree v3.4.0	Chen et al.^48^	https://otree.readthedocs.io
oTree v5.11.1	Chen et al.^48^	https://otree.readthedocs.io
Python v3.7.9	Python Software Foundation	https://www.python.org
R v4.2.1	R Core Team	https://cran.r-project.org
ChatGPT-3.5	OpenAI	https://platform.openai.com/docs/models/gpt-3-5
ChatGPT-4	OpenAI	https://platform.openai.com/docs/models/gpt-4

**Other**		

Experiment 1 pre-registration	AsPredicted	https://aspredicted.org/Q3R_RYX
Experiment 2 pre-registration	AsPredicted	https://aspredicted.org/B7W_YB9

### Experimental model and study participant details

#### Experiment 1

The experiment was approved by the ethics committee of the Institute of Psychology at Leiden University (2023-10-09-C.K.W. de Dreu-V1-5011) and did not involve deception. Participants were residents of the United Kingdom and recruited via Prolific (*n* = 252, 48.81% were female, self-reported gender). Participants were between 18 and 70 years of age (*M* = 30.10, *SD* = 9.45), provided informed consent, and received full debriefing after participating. They received a standard fee of £5.00 and their decisions were fully incentivized (*M* = £2.08, *SD* = 0.90, range: £0.30–3.60). Participation took approximately 30 min.

#### Experiment 2

The experiment received ethics approval from Leiden University (2022-11-24-C.K.W. de Dreu-V1-4365) and did not involve deception. The experiment was programmed in oTree (version 3.4.0)^48^ and written in Python (version 3.7.9). Participants (*n* = 312) were between 17 and 44 years of age (*M* = 21.51, *SD* = 3.95; 74% were female, self-reported gender), provided informed consent, and received full debriefing after participating. They received a standard fee of €5.00 and their decisions were fully incentivized (*M* = €7.50, *SD* = €2.29, range: €2.50–14.79; details are given below). The experiment took approximately 60 min. No participants were excluded from analyses.

### Method details

#### Archival analysis

To quantify how often leaders justify their resort to war with claims of self-defense, we used the war manifestos database.^25^ This database contains 261 declarations of war issued between 1508 and 1941 by a sovereign entity and directed against another sovereign entity that were, when issued, public. Accordingly, war manifestos pertain to interstate warfare and are publicly available documents created to persuade not only the issuing leader’s own followers, but also external audiences like (potential) allies whose support may need to be secured or reinforced and enemy troops who might be swayed into questioning their own government’s motives.^26^ In addition, war manifestos offer explicit justifications for the decision to go to war at its outset, when motives and positions may still be ambiguous. Hathaway and colleagues^25^ coded these articulated reasons for instigating hostilities into the following twelve categories of common just war claims: 1) enforcement of inheritance laws, succession rules and other hereditary rights; 2) self-defense or repelling aggression; 3) balance of power concerns; 4) declaration of independence; 5) tortious wrongs; 6) collection of debts; 7) protection of trade interests; 8) protection of diplomatic relations; 9) humanitarian considerations; 10) religious claims; 11) violation of a treaty obligation; and 12) other reasons. We identified category 2 as ‘non-revisionist’ defense reasons.

Because we are interested in publicly stated motives at the onset of war, we excluded counter-manifestos (documents responding to an initial manifesto) and quasi-manifestos (documents that met all but one of the criteria for a war manifesto^26^). Next, we identified for each issuing entity whether their main aim for staging war was revisionist (‘attack’) or non-revisionist (‘defense’). Following prevailing definitions of asymmetric conflict and warfare in political science and international relations,^6^^,^^36^ we assigned a revisionist aim when the issuing entity initiated and carried out offensive military operations to achieve strategic objectives like expanding control over territory (i.e., ‘attack’), regardless of whether the operation was preemptive, preventive, or opportunistic. We assigned a non-revisionist aim when the issuing entity responds to aggressive actions by another sovereign entity to protect its territory, interests, or assets (i.e., ‘defense’). This ‘first-mover’ coding rule ensures consistency and avoids relying on post hoc interpretations of leaders’ motives.

For declarations of war issued between 1816 and 2014 we adopted the classification of (non)revisionist status from the fifth version of the Militarized Interstate Disputes (MID) database from the Correlates of War project.^36^ For older manifestos (prior to 1816), and for manifestos not covered in the MID database, we employed a structured multi-step process to ensure consistency and transparency in coding. A research assistant, who was blind to hypotheses and the content of the manifestos, consulted historical sources and peer-reviewed articles to classify the issuing entity as revisionist or non-revisionist. The research assistant recorded the classification and source in a codebook. Unclarities were discussed within the research team, with a consensus classification for 237 of the 261 manifestos; 120 were designated as revisionist attackers and 117 as non-revisionist defenders. For the remaining 24 manifestos, no consensus could be reached and these were excluded from final analyses. The war manifestos database is publicly available through Hathaway et al.^25^^,^^26^ Our codebook, including each war manifesto’s actual revisionist or non-revisionist status and a reference to a peer-reviewed journal article for each classification, is available in an OSF repository (https://osf.io/DE34J/).

#### Experiment 1

Participants were instructed that they would be shown short excerpts from speeches of country leaders in which they talk about an upcoming or ongoing conflict with another country. Some excerpts were from leaders of an ‘attacker’ country, and some were from leaders of a ‘defender’ country (for the purpose of clarity, we used ‘attacker’ for revisionist and ‘defender’ for non-revisionist countries; Figure 2A). Instructions defined attacker (“this is the party in a conflict or war that initiates and carries out offensive military operations to achieve strategic objectives like expanding control over territory”) and defender (“this is the party in a conflict or war that responds to an attacker in order to protect its territory, interests, or assets”). To ensure that participants understood the difference between attackers and defenders, they answered three practice questions. Only after all practice questions were answered correctly, participants could continue with the main task.

To optimize participants’ engagement while minimizing cognitive load, each participant was presented with a subset of 12 excerpts. To ensure a balanced representation, the 36 excerpts were initially shuffled randomly and then divided into three sets, with each set containing an equal distribution of speech excerpts from six revisionist attackers and six non-revisionist defenders. Excerpt presentation within each set happened in a random order.

They were asked to carefully read and assess the content of each excerpt, and to decide whether it belongs to the leader of an attacker or defender country. For each correct identification they would receive £0.30 (on average participants correctly identified 57.8% of the excerpts, for an extra average earning of £2.08 [*SD* = 0.90, range: £0.30–3.60]).

After classifying each speech excerpt, participants were asked to rate their confidence in their classification and to indicate their support for the leader to whom the speech excerpt belongs (1 = not at all confident/not at all, to 7 = extremely confident/very strongly). Participants were only able to submit their classification, confidence rating, and support rating after 20 s, to make sure they spent sufficient time on each excerpt. On average, participants spent 63.89 seconds per speech excerpt (*SD* = 93.47, range: 20.41–3214.32).

The 36 war speech excerpts were obtained from the Justifications of War Database,^47^ which catalogs primary-source justifications of war from various historical periods. A research assistant, who was blind to the study hypotheses, conducted the initial screening process. During this process, speeches were included only if (a) they related to interstate conflicts (excluding internal uprisings or civil wars where revolutionary forces might simultaneously hold positions of attacker and defender), (b) were complete and publicly accessible, and (c) contained public statements by leaders aimed at their followers. From each selected speech, we extracted only the first reason provided for justifying the conflict. This approach aimed to control for length and cognitive load and reduced the risk of selectively choosing more persuasive arguments. From each eligible speech excerpt, the research assistant removed any contextual identifiers (e.g., country names, nationalities, dates, locations, titles) so that readers could not link an excerpt to any specific conflict, historical period, or leader.

The research team then reviewed these decontextualized excerpts to confirm that each clearly referenced (a) a military action in an international conflict, (b) contained correct and complete decontextualization ensuring no excerpt could be linked back to a specific conflict, and (c) referenced a position consistent with a revisionist attacker or non-revisionist defender state. With regard to (c), we used the fifth version of the Militarized Interstate Disputes (MID) database from the Correlates of War project^36^ to classify issuing states as revisionist attacker, or as non-revisionist defender. For speeches that were from before 1816, and for speeches we could not classify using the Correlates of War project, we used peer-reviewed articles to determine their position.

This left us with 38 eligible war speech excerpts. To achieve a more balanced distribution by geography and historical context, and to ensure an equal number of revisionist and non-revisionist excerpts, we removed two speeches from Great Britain. The final pool consisted of 18 excerpts from revisionist attacker states and 18 excerpts from non-revisionist defender states, representing 17 different countries across all continents except Oceania. The speeches spanned a historical range from 1754 to 2008. Full details on each selected excerpt, including source information, classification, and the supporting references for its coding, are available in Methods S2 and in the OSF repository (https://osf.io/DE34J/).

After evaluating all excerpts, participants were asked for their gender and age, indicated their familiarity with historical events involving military disputes (ranging from “not at all familiar” to “I have an expertise in this area”), and indicated whether they were able to assign any specific conflicts to any of the excerpts (ranging from 0 to 12 excerpts). Finally, we asked participants for open feedback (not analyzed).

To determine serious participation, we included three attention checks and notified participants that failing two out of three attention checks would exclude them from data analysis (as pre-registered). No participant missed more than one attention check, so we did not exclude any participants from the final analyses following our pre-registered exclusion criterion.

#### Experiment 2

Upon registering for the experiment, participants filled out an online survey (for details see Methods S3). One to two weeks later, participants came to our decision laboratory in groups of six. We ensured that participants were unacquainted and prohibited interaction prior to the experiment. Upon arrival, participants were randomly assigned to one of the individual cubicles within the laboratory and given written instructions for the intergroup attacker-defender game.^6^ The experimental instructions used neutral language throughout (e.g., revisionist attacker and non-revisionist defender positions were referred to as Role A and Role Z, leaders were labeled coordinators, followers were labeled members, and terms like ingroup defense and outgroup aggression were avoided). After the rules of the task were explained, participants answered 21 practice questions to probe their understanding of the task. Only after all practice questions were answered correctly, participants could proceed with the experiment.

For the main task, participants were divided in two three-person groups, each consisting of one leader and two followers. At the beginning of each contest round, leaders were informed whether their group was in the attacker or defender position for that round. Crucially, leaders were then asked to convey this position (attacker or defender) to their followers. In the baseline ‘no-deception’ treatment, this information always had to be truthful. In the experimental ‘deception’ treatment, this information could be truthful or not (i.e., “you can give the information that you think is best”). Groups were in each position on half of the rounds. To ensure unpredictability in position assignment, we implemented a non-systematic sequence of randomly assigned positions (consistent across groups and treatments), with the constraint that groups could not have the same role for more than four consecutive rounds. Participants were only instructed that they would be in the position of attacker in some rounds and in the position of defender in others, but they were not told the exact probabilities.

In every round, leaders and followers each received an endowment of 20 resources. Followers were informed of the position their leader had communicated and then decided how much of their endowment to contribute to their group’s conflict pool (*x*) versus how many to keep (*k*). Followers in the (actual) attacker group invested in outgroup attack (*x*_A_), while those in the (actual) defender group invested in ingroup defense (*x*_D_). Simultaneously, leaders indicated the average number of resources they expected their followers to contribute to the conflict pool to probe beliefs (leaders earned €0.10 for each correct expectation).

Followers’ contributions to the conflict pool were non-recoverable and, hence, wasted. However, if attackers collectively invested more in conflict than the defenders (*x*_A1_ + *x*_A2_ > *x*_D1_ + *x*_D2_), the followers of the attacker group won the remaining resources of the followers of the defender group (i.e., *k*_D1_ + *k*_D2_). These ‘spoils of war’ were divided equally among the attacker followers and added to their remaining endowments, while defenders earned nothing. Otherwise (i.e., in the case of *x*_A1_ + *x*_A2_ ≤ *x*_D1_ + *x*_D2_), defenders ‘survived’, and all followers simply kept their remaining endowments (*k*). If the attackers succeeded, their leader appropriated the resources of the defending leader. When defenders succeeded, their leader appropriated the resources of the attacking leader. Thus, contributions in attacker groups represented outgroup aggression (trying to take resources from the defender group), while those in defender groups reflected ingroup defense (trying to prevent attackers from taking their remaining resources).

At the end of each round, followers were informed about the total contribution both groups made to their conflict pool and how much they earned given the position that their leader communicated, not their actual role. For example, if a group was assigned to the defender role but the leader falsely communicated that they were in attack, followers received feedback as if they were attackers. This prevented followers from inferring their group’s true position (attacker or defender). Leaders received full information and were also informed about the earnings of both their own group’s followers and those of the opposing group, given each group’s ‘true’ position. This completed one round. The task consisted of 20 rounds in total.

Participants were paid out based on the average of 4 randomly selected rounds (participants could maximally earn €8.00). On average, participants earned €2.59 (*SD* = 1.66, range: €0.00–8.00). Leaders could also earn a bonus if they correctly predicted their followers’ contributions (leaders earned €0.10 for each correct expectation and could maximally earn €2.00). Participants received, on average, €0.32 (SD = 0.22, range: €0–1.00).

Following the intergroup attacker-defender contest, participants filled in a questionnaire measuring their ingroup identification (i.e., the solidarity and satisfaction scale^49^). Thereafter, participants were asked how many rounds they thought their group was in attack and defense. In the deception treatment, participants were also asked on how many rounds they believed their leader honestly communicated their position. These questions were also incentivized (i.e., participants received €0.50 per correct expectation). Participants, on average, earned €0.44 (SD = 0.54, range: €0.00–1.50) for this task. In both treatments, followers were furthermore asked whether they would vote for their leader to be re-elected (yes/no). Thereafter, participants rated the social appropriateness of all possible forms of leader communication: leaders in an attacking position communicating that their group was in an attacking position, leaders in an attacking position communicating that their group was in a defensive position, and so on. Finally, followers received 10 additional resources which they could use to reward or punish their respective leader. That is, leaders either gained or lost money based on the level of endorsement they received from their followers. Leaders received, on average, €0.49 (SD = 0.34, range: €-0.50–1.00). This completed the experiment. Participants were then informed about their earnings and received a debriefing.

### Quantification and statistical analysis

Statistical models were fitted using the lme4 package in R.^50^ Multilevel (logistic) models included random intercepts for participants nested within their group to account for violations of independence, since participants made repeated decisions and were part of a group in which they potentially influenced each other’s decisions over time. All reported statistical tests were two-tailed.

#### Archival analysis

We conducted two binomial tests to assess how frequently war manifestos issued by attackers and defenders contained the claim ‘self-defense, repelling aggression’ (Table S1) and fitted a logistic regression to examine how often attacker war manifestos included ‘self-defense, repelling aggression’ as a reason to resort to war throughout history (Table S2).

#### Experiment 1

We fitted multilevel (logistic) regression models to examine how the predicted and ‘true’ position of a leader impacted participants’ support for this leader’s causes (Table S3), how accurately participants classified leader speeches as either attack or defense motivated (Table S4), how the predicted and ‘true’ position of a leader impacted ChatGPT’s ‘trust’ for this leader’s causes (Tables S5 and S6), and how accurately ChatGPT classified leaders' speeches as either attack or defense (Tables S7 and S8).

#### Experiment 2

We fitted multilevel (logistic) regression models to examine how one’s ‘true’ position in the no-deception treatment impacted followers’ contributions to conflict (Table S9), free-riding (Table S10), conflict success (Table S11), and earnings (Table S12). We also fitted multilevel (logistic) regression models to examine how treatment impacted followers’ contributions to conflict (Table S13) and how one’s ‘true’ position in the conflict impacted leader deception (Table S14). Furthermore, we fitted multilevel (logistic) regression models to examine how attacking leader (dis)honesty impacted their expectations about their followers’ contributions to conflict (Table S15), followers’ actual contributions to conflict (Table S16), free-riding (Table S17), conflict success (Table S18), and the earnings of attacking leaders (Table S20) and followers (Table S22). We also fitted multilevel (logistic) regression models to examine how treatment impacted attackers’ conflict success (Table S19) and attackers’ earnings (Table S21). Finally, we fitted a multilevel regression model to examine how leader (dis)honesty and their followers’ earnings impacted how many points followers donated to their leader (Table S23).

### Additional resources

#### Open Science Framework

Full codebooks, de-identified data, and supplemental information for all studies are publicly available (https://osf.io/DE34J/).

#### Pre-registrations

##### Experiment 1

We pre-registered the experimental design, analysis plan, sample size, and exclusion criteria via AsPredicted (on November 30^th^, 2023, https://aspredicted.org/Q3R_RYX). No participants were excluded from the analyses. As pre-registered, we also collected an expert sample. However, only 14 of 91 invited scholars participated (see Methods S4 for details on the recruitment procedure). Preliminary findings suggest that experts who completed the survey were significantly better at detecting false narratives and classifying defense and attack positions with less bias. While this provides tentative evidence that knowledge may help pre-empt deception, the small sample size and possibility of self-selection bias prohibit more conclusive statements, and further studies are needed to robustly test these possibilities.

##### Experiment 2

We pre-registered the experimental design, analysis plan, sample size, and exclusion criteria via AsPredicted (on January 31^st^, 2023, https://aspredicted.org/B7W_YB9). There were no deviations from our pre-registration and results confirmed all pre-registered hypotheses.
