# Supplementary material for: How defense rhetoric escalates intergroup conflict
Source: iScience. 2025 Nov 5;28(11):113749. doi: 10.1016/j.isci.2025.113749 (PMC12682265; doi:10.1016/j.isci.2025.113749)
Supplement: Document S1. Tables S1–S24 and Methods S1–S4 [file mmc1.pdf]

iScience, Volume *28*

Supplemental information

How defense rhetoric escalates intergroup conflict

Luuk L. Snijder, Jörg Gross, and Carsten K.W. De Dreu

Methods S1

Estimating death toll from armed conflict

For all available countries included in the Uppsala Conflict Data Program (UCDP GED; V24.6, 2024), we selected the period from 2010 to 2023 and computed the sum of deaths from direct acts of armed conflict (best estimate provided). The derived sum thus excludes mortality from conflict-induced famine and disease, and likely is a conservative estimate of the lethality of human conflict.

Reference

Sundberg, R. & Melander, E. (2013). Introducing the UCDP georeferenced event dataset. *J. Peace Res. 50*, 523–532.

Methods S2

Speech excerpts

Each participant was presented with a subset of 12 speech excerpts. To ensure a balanced representation, the 36 excerpts were initially shuffled randomly and then divided into three sets, with each set containing an equal distribution of speech excerpts from six revisionist attackers and six non-revisionist defenders. Full details about the speeches—including their titles, specific events, the leaders' position, issuing countries, years, links to the speeches, and original sources—are available in an OSF repository (https://osf.io/DE34J/). The studies, which were programmed in Qualtrics, can also be accessed in the same repository. Below are the excerpts presented to participants.

Set 1.

E1.1. "[…] The [enemy's] Government has begun the war to make you who did not die share the fate of those who did, to destroy your freedom by abasing the well-known bravery of [our] sons which is recorded in world history, to invade your patrimony and your houses, to acquire as slaves your old parents, to make exiles of your sons, and to reckon [our] heroes as if they did not exist, and to attack with its army in the north and in the south. […]"

E1.2. "[…] My government will exhaust all peaceful avenues to find a solution to the territorial problem – a solution that is just, realistic and worthy of the two countries – and it will do so in a concrete manner, given the incidents that have been taking place along the southeastern border. However, complying with my obligations towards the country and [our] people and in keeping with [our] firm aspiration for peace, today I have had to declare a state of emergency and national mobilization. The cabinet ministers and I signed this decree today. Citizens, I ask you to maintain the calm and unity that reflect favourably upon you, as well as the absolute determination to defend our country's integrity and honour. […]"

E1.3. "[…] To our proposals to resolve these issues of relations in a peaceful, civilized way we were responded to with tanks, planes, guns, murders, robberies. And it just shows the true essence of the current [enemy] leadership. Finally, it can no longer hide behind his characteristic hypocrisy. It has removed a terrible mask. […]"

E1.4. "[…] Our objective, endorsed by all sides of the House in recent debates, is that the people of the [disputed area] shall be free to determine their own way of life and their own future. The wishes of the [those people] must be paramount. But they cannot be freely expressed, let alone implemented, while the present illegal occupation [by the enemy] continues. […]"

E1.5. "[…] The knavish mass of the […] barbarians, daring to perpetrate such outrage and to capture our city and kill our officials, really arouse the wrath of the deities as well as men. It is impossible to suffer their violence to grow. They must be quickly extirpated, in order to make clear the laws and statutes of our empire. […]"

E1.6. "[…] For months we have been suffering under the torture of a problem which the [enemy's doing] created - a problem which has deteriorated until it becomes intolerable for us. [city XY] was and is [our] city. The Corridor was and is [ours]. Both these territories owe their cultural development exclusively to [our] people. [city XY] was separated from us, the Corridor was annexed by [the enemy]. As in other [of our] territories of the East, all [our] minorities living there have been ill-treated in the most distressing manner. […]"

E1.7. "[…] Execution of these Assurances, and of the Treaties on which they were founded, has been evaded under the most frivolous Pretences; and the unjustifiable Practices of the [enemy's] Governors, and of the Officers acting under their Authority, were still carried on, till, at length, in the Month of April, One thousand seven hundred and fifty four, they broke out in open Acts of Hostility, when, in Time of profound Peace, without any Declaration of War, and without any previous Notice given, or Application made, a Body of the [enemy's] Troops, under the Command of an Officer bearing [the enemy's] King's Commission, attacked in a hostile Manner, and possessed themselves of [our] Fort […]."

E1.8. "[…] Last night, [the enemy] troops committed what amounts to an act of aggression against [our] peacekeepers and the civilian population in [country XY]. What took place is a gross violation of international law and of the mandates that the international community gave [us] as a partner in the peace process. […]"

E1.9. "[…] The people of [our nation] and our friends and allies will not live at the mercy of an outlaw regime that threatens the peace with weapons […]. We will meet that threat now, with our Army, […], so that we do not have to meet it later with armies of fire fighters and police and doctors on the streets of our cities. […]"

E1.10. "[…] We do not attack anyone. On the contrary, in a number of cases we are protecting the lives and dignity of people, our citizens and foreign citizens, through our presence as peacekeepers in different countries […]. I think it is absolutely clear to you that if someone thinks they can kill our citizens in impunity, kill our soldiers and officers who are peacekeepers, this is something we can never accept. Anyone who tries such a thing will meet with a crushing response. […]"

E1.11. "[…] We have already said that there are only three ways left to [our nation] to escape from the pressure of surplus population. We are like a great crowd of people packed into a small and narrow room, and there are only three doors through which we might escape, namely, emigration, advance into world markets, and expansion of territory. The first door, emigration, has been barred to us by the anti-[us] immigration policies of other countries. The second door, advance into world markets, is being pushed shut by tariff barriers and the abrogation of commercial treaties. What should [our nation] do when two of the three doors have been closed against her? It is quite natural that [our nation] should rush upon the last remaining door. […]"

E1.12. "[…] Soldiers, officers, NCOs, all the deprived people, rise to save your country. Rise up against the foreign mercenaries, the plundering, murderous [enemy]. You have sensitive moments in front of you. Join our army and crush the traitors of your country. Your uprising will save millions of [us] from oppression and despotism. Rise, do not allow the treacherous [enemy] to flee. Arrest them and hand them over to your brothers in the armed forces and to the liberators of [our nation] so that they pay for their crimes and treason. […]"

Set 2.

E2.1. "[…] We have always complied, even with what is illegitimate and unjust among the demands and allegations of the evil ones, in the hope that the world would awake and lift the sanctions against our people and so that we avoid the evils of war. And after they ran out of excuses or cover, the invading aggressors came openly and shamelessly with vicious intent as we know them and their intentions to be. O brothers, you know that our country's policy is to avoid evil. But when evil comes, armed with deceit and destruction, we must face them with faith and holy struggle in a manner which dignifies us and satisfies God. […]"

E2.2. "[…] [The enemy] has for centuries pursued the aim of rendering the peoples of [our continent] defenceless against [their] policy of world conquest by proclaiming a balance of power, in which [they] claimed the right to attack on threadbare pretexts and destroy that State which at the moment seemed most dangerous. […]"

E2.3. "[…] The motive of the present war is to resist the aggression of [the enemy's] imperialism, to save the country from danger and extinction and, at the same time, to expedite the task of national reconstruction. [The enemy's] imperialism seeks, politically, to divest [our nation] of her independence and freedom and, economically, peg her down to a position of permanent productive backwardness and make her a commercial vassal state of [the enemy's nation]. The present danger is, therefore, incomparable to any of the military and political setbacks which [we] experienced in the past. […]"

E2.4. "[…] There is no end to the humiliation and insults that they have heaped upon us. To think about it makes the heart sick. Since the […] incident on [date], the more indignities we have borne the more we have yielded, the more violent has [the enemy's] oppression become. Give them an inch and they take a foot. Now we have reached the point when we can endure it no longer; we will give way no more. The whole nation must rise as one man and fight these bandits until we have destroyed them, and our own life is secure. […]"

E2.5. "[…] [Our nation], within and beyond the Confederation, has come to the conclusion that, in preferring these demands, the Government [of the enemy] wished to subject us to a humiliation which the country cannot endure; and that, contrary to the desire and intentions of [our nation], war has been forced on us by [the enemy]. […]"

E2.6. "[…] The further aggravation of the situation in [country XY] affects the vital interests of [us] and other States [of the same political system/beliefs], and the security interests of the States of [our] community. The threat to [our political] system in [country XY] at the same time constitutes a threat to the foundations of peace in [our continent]. […]"

E2.7. "[…] [Our nation] has just been the object of a violent and premeditated attack, which is an insolent defiance of the law of nations. Before any declaration of war had been sent to us, even before the [enemy's] Ambassador had asked for his passports, our territory has been violated. [Our] Empire has waited till yesterday evening to give at this late stage the true name to a state of things which it had already created. […]"

E2.8. "[…] It is not enough to have expelled the barbarians who have bloodied our land for two centuries; it is not enough to have restrained those ever-evolving factions that one after another mocked the spectre of liberty that [the enemy] dangled before you. We must, with one last act of national authority, forever assure the empire of liberty in the country of our birth; we must take any hope of re-enslaving us away from the inhuman government that for so long kept us in the most humiliating torpor. In the end we must live independent or die. […]"

E2.9. "[…] In view of the present international situation and its possible future developments, and in light of the serious possibilities threatening the [our] sovereignty and national security, on the one hand, and world peace and security, on the other; and in response to the demands of national responsibility to [our] nation's land, culture and heritage; and in accordance with the principles of the Non-aligned Movement, [our nation] feels called upon to take the initiative by issuing the following declaration. […]"

E2.10. "[…] Brothers, when we talk about [the opposing group] we are not talking about individuals in our society who hate [our culture] and [our nation]. We are talking about the influence of the great powers and attempts by the great powers to harm your revolution, great country, heritage, beliefs and glories. They believe they can take [our nation] back to its former state of poverty and humiliation. […]"

E2.11. "[…] The nation of [the enemy], our brotherly nation, under the jackboots of the fascists, is looking for an opportunity to rise against the puppet satan. What better opportunity for us to make of the present situation against the forces of an aggressive government which has no excuse for what it has done against our revolution during the past year; and we shall certainly make use of this opportunity. […]"

E2.12. "[…] If [our country] in former centuries silently bore such outrages on her rights and honour, she only did so because, disunited as she was, she did not know her strength. Now, when the ties of an intellectual and constitutional union, first knit by the war of liberation, are drawing the various States of [our country] more and more closely together – now, when the defences of our country leave no loophole for a foreign foe to creep in – [Our country] has both the will and the power to repel the renewed insults of [the enemy]. […]"

Set 3.

E3.1. "[…] We are at the moment suffering from the invasion of a cruel foe; an invasion carried out with unparalleled ferocity. The nation today is engaged in a bitter struggle. All our people are endeavoring by sacrifice of flesh and blood – yea, of their very lives – to secure the survival of the State. In this War of Resistance, the warm patriotism of our fellow-[people] overseas, and the courage and enthusiasm of all our citizens is more than enough to inspire and encourage us, and to convince us that out of this period of trial and suffering, we shall be able to achieve the revival of our nation. […]"

E3.2. "[…] But [the enemy], which for a long time has shown an unconcealed desire to acquire our country, now prepares to attack us…The hour is grave. Arise, each of you. Take up arms, and rush to the defence of your country. Rally to your chiefs; obey them with single purpose, and repel the invader. […]"

E3.3. "[…] [Our people] picked up the gauntlet with no other object than that of defending their independence, which cost our forefathers so much blood and which is so dear to us, to the uttermost. Thanks to the Almighty, our arms were blessed in a manner which not only struck the world with amazement, but far exceeded our own expectations. Although the capture of [one of our leaders] and the occupation of [city XY] were heavy blows to us, I am nevertheless glad to be able to say that our [people] are still full of courage and determined to continue to fight for the preservation of our dearly purchased independence, and, if necessary, like so many of our dear ones, to die as brave and never-to-be-forgotten heroes. […]"

E3.4. "[…] With patience we have faced the fact that, under the pretense that [our nation] was desirous of war, enmity has been awakened against us in the East and the West, and chains have been fashioned for us. The wind then sown has brought forth the whirlwind which has now broken loose. We wished to continue our work of peace, and, like a silent vow, the feeling that animated everyone from the Emperor down to the youngest soldier was this: Only in defense of a just cause shall our sword fly from its scabbard. The day has now come when we must draw it, against our wish, and in spite of our sincere endeavors. [The enemy] has set fire to the building. We are at war with [Enemy 1] and [Enemy 2] — a war that has been forced upon us. […]"

E3.5. "[…] [Our nation] is determined to enforce the demands of the Security Council by confronting the grave and growing danger of [the enemy's leader] and his weapons […]. This [leader] will not be allowed to intimidate and blackmail the civilized world, or to supply his terrible weapons to terrorist groups, who would not hesitate to use them against us. The safety of [our] people depends on ending this threat. […]"

E3.6. "[…] If, however, the efforts of nations and those we are making are in vain, and if diabolical force prevails, bringing about a war which spreads unhappiness and misery on human beings, [our nation] will uprise, her Emperor at the head, and her people, whose heroism is well known, confident in Divine aid, will defend themselves against the invader to the last drop of their blood, protecting themselves in the natural fortresses, the mountains and deserts, which the Lord has given her. […]"

E3.7. "[…] A momentous hour has struck for [us]. Envious rivals everywhere force us to legitimate defense. The sword has been forced into our hands. I hope that in the event that my efforts to the very last moment do not succeed in bringing our opponents to reason and in preserving peace we may use the sword, with the help of God, so that we may sheathe it again with honor. […]"

E3.8. "[…] Yes, it was the [enemy] who broke the peace with unprovoked aggression. They are on [our] sovereign territory and there are [our] people under the heel of the [enemy]. We sent the task force to rectify that situation. We hope to do so by all peaceful means and shall continue to try to do so. In the meantime, our first duty must be to protect our boys. […]"

E3.9. "[…] [Our nation] has no territorial intentions and accordingly [our] actions with regard to [the enemy] do not violate existing treaties in any way. The present situation originated in an unwarranted attack made by [the enemy's] forces on [our] garrison troops legitimately stationed in [their country]. [Our nation] did everything in her power to reach a local settlement of the incident, even at the sacrifice of strategical advantage. […]"

E3.10. "[…] The Government will pursue in the spirit of international justice a national policy embodying the unanimous will of the nation in order to secure the stability of [our area] and promote the common prosperity of all nations and brighten thereby the horizon of our international relations. As regards to [the enemy's country], it is to be deeply regretted that despite our efforts in the past to act in concert with their country for the purpose of ensuring stability in [our area], the [enemy's] Government have as yet failed to understand fully our true intentions, and there have arisen a number of problems between the two countries. […]"

E3.11. "[…] Here is not just an army marching toward a military objective, but a whole people, forty-four million souls, against whom the blackest of all injustices has been committed – that of denying them a place in the sun. […] To military sanctions we shall reply with military measures! To acts of war we shall reply with acts of war! […]"

E3.12. "[…] After arbitrarily violating the rights of [two of our leaders], disturbing and overthrowing good order and legitimate government in the interior of the realm, committing against the sacred person of [the king and his family] outrages and brutalities which continue to be renewed daily, those who have usurped the reins of government have at last completed their work by declaring an unjust war on [another leader] and attacking his provinces situated in [his area]. Some of the territories of [ours] have been affected by this oppression, and others have only escaped the same fate by yielding to the threats of the dominant party and its emissaries. […]"

Methods S3

Online Survey

Participants provided informed consent and were instructed that their decisions, and those of other participants, would influence both their own payment and that of others. Participants completed the following tasks in a random order: (i) the social value orientation slider measure[1], (ii) the rule-following task[2], (iii) the coin-toss task[3], and (iv) the social dominance orientation questionnaire[4]. In the social value orientation slider measure, participants decide how to allocate points between themselves and an unknown other person. To incentivize decisions, participants were randomly paired twice with another participant in their group, with each participant once being selected as the allocator and once as the receiver. On average, participants earned €1.28 (*SD* = 0.03, range: €1.21–1.34). In the rule-following task, participants place 30 balls in one of two buckets. Whereas one bucket generates more income than the other (10 vs. 5 cents per ball), participants are given the rule to place the balls in the less profitable bucket. The number of balls in the less profitable bucket serves as a measure of (arbitrary) rule following. The task was incentivized, and participants earned on average €2.00 (*SD* = 0.56, range: €1.50–3.00). In the coin-toss task, participants predict the outcome of 15 computerized random coin flips. Before each coin flip, they are asked to make a prediction in their mind (heads or tails). After they are shown the outcome of the coin flip, they are asked to indicate whether their prediction was correct. For each correct prediction, they earn €0.10. On average, participants earned €0.95 (*SD* = 0.25, range: €0.30–1.50). Finally, to measure social dominance orientation, participants respond to eight items measuring one's degree of preference for inequality among social groups (not incentivized). After completing these four tasks, participants filled in their demographics (gender, age, and political orientation) and were informed that they would receive information about their earnings in these tasks after they completed the experiment.

References

1. Murphy, R.O., Ackermann, K.A. & Handgraaf, M.J.J. (2011). Measuring social value orientation. *Judgment Decis. Mak. 6*, 771–781.
2. Gross, J. & De Dreu, C.K.W. (2021). Rule following mitigates collaborative cheating and facilitates the spreading of honesty within groups. *Pers. Soc. Psychol. Bull. 47*, 395–409.
3. Van Breen, J.A., De Dreu, C.K.W. & Kret, M.E. (2018). Pupil to pupil: The effect of a partner's pupil size on (dis)honest behavior. *J. Exp. Soc. Psychol. 74*, 231–245.
4. Pratto, F., Sidanius, J., Stallworth, L.M. & Malle, B.F. (1994). Social dominance orientation: A personality variable predicting social and political attitudes. *J. Pers. Soc. Psychol. 67*, 741–763.

Supplemental Tables

Archival analysis

We conducted two binomial tests to assess how frequently war manifestos issued by attackers and defenders contained the claim `self-defense, repelling aggression` (Table S1). Specifically, we tested whether the proportion of self-defensive claims deviated significantly from 0.5. In the first test, we examined the use of self-defensive claims in war manifestos issued by attackers. In the second test, we examined the use of self-defensive claims in war manifestos by defenders.

Table S1. Binomial tests for claims of self-defense in war manifestos issued by attackers and defenders.

	success	n	p	proportion	95% CI
attack	77	120	0.002	0.642	[0.55, 0.73]
defense	94	117	< 0.001	0.803	[0.72, 0.87]

We fitted a logistic regression to examine how often attacker war manifestos included 'self-defense, repelling aggression' as a reason to resort to war throughout history (Table S2). In this model we coded if war manifestos issued by attackers included the claim 'self-defense, repelling aggression' (= 1) or not (= 0) as the dependent variable, and the year as predictor.

Table S2. Logistic regression modeling how often war manifestos issued by attackers used self-defense as a reason to resort to war throughout history.

		Estimate	SE	z	p	95% CI
**Model**	intercept	-7.52	3.27	-2.30	0.021	[-14.24, -1.34]
	year	0.005	0.002	2.48	0.013	[0.001, 0.01]

Experiment 1

Multilevel (logistic) models included random intercepts for participants to account for violations of independence, since participants made repeated decisions. All reported statistical tests were two-tailed.

We fitted a multilevel regression model to examine how the predicted and 'true' position of a leader impacted participants' support for this leader's causes (Table S3). In this model with leader support (on a scale from 1-7) as the dependent variable, we included the predicted position (0 = revisionist attacker, 1 = non-revisionist defender) and 'true' position (0 = revisionist attacker, 1 = non-revisionist defender) of the leader as fixed factors.

Table S3. Mixed effects regression modeling how the predicted and 'true' position of a leader impacted support for their causes.

		Estimate	SE	t	p	95% CI
**Model**	intercept	2.70	0.08	34.60	< 0.001	[2.55, 2.85]
	actual defender	0.05	0.05	1.01	0.314	[-0.04, 0.13]
	predicted defender	1.92	0.05	39.60	< 0.001	[1.82, 2.01]

We fitted two multilevel binomial regression models to assess how accurately participants classified leader speeches as either attack or defense motivated (Table S4). Specifically, we tested whether the proportion of correct classifications deviated significantly from chance (0.5). In the first model, we examined the correct classification of attacker speeches. In the second model, we examined the correct classification of defender speeches.

Table S4. Multilevel binomial tests for classifying attacker and defender speeches.

		Estimate	SE	z	p	95% CI
**Model**	attack	-0.17	0.06	-2.81	0.005	[-0.28, -0.05]
	defense	0.85	0.06	13.19	< 0.001	[0.73, 0.98]

We fitted two multilevel regression models to examine how the predicted and 'true' position of a leader impacted ChatGPT's 'trust' in this leader's causes (Table S5 and S6). In both models with leader trust (on a scale from 1-7) as the dependent variable, we included predicted position (0 = revisionist attacker, 1 = non-revisionist defender) and 'true' position (0 = revisionist attacker, 1 = non-revisionist defender) as fixed factors. The first model only included data of ChatGPT3.5. The second model only included data of ChatGPT4.

Table S5. Mixed effects regression modeling how the predicted and 'true' position of a leader impacted ChatGPT3.5's trust in their causes.

		Estimate	SE	t	p	95% CI
**Model**	intercept	3.56	0.04	85.41	< 0.001	[3.48, 3.64]
	actual defender	-0.08	0.05	-1.81	0.070	[-0.17, 0.01]
	predicted defender	1.10	0.05	23.31	< 0.001	[1.01, 1.19]

Table S6. Mixed effects regression modeling how the predicted and 'true' position of a leader impacted ChatGPT4's trust in their causes.

		Estimate	SE	t	p	95% CI
**Model**	intercept	3.38	0.09	38.58	< 0.001	[3.21, 3.55]
	actual defender	0.34	0.10	3.39	0.001	[0.14, 0.54]
	predicted defender	1.01	0.10	9.88	< 0.001	[0.81, 1.22]

We conducted four binomial tests to assess how accurately ChatGPT classified leaders' speeches as either attack or defense (Table S7 and S8). Specifically, we tested whether the proportion of correct classifications deviated significantly from chance (0.5). In the first test, we examined the correct classification of attacker speeches. In the second test, we examined the correct classification of defender speeches. We ran these tests separately for ChatGPT3.5 and ChatGPT4.

Table S7. Binomial tests for ChatGPT3.5 attacker and defender speech classifications.

	success	n	p	proportion	95% CI
attack	314	720	0.001	0.44	[0.40, 0.47]
defense	492	720	< 0.001	0.68	[0.65, 0.72]

Table S8. Binomial tests for ChatGPT4 attacker and defender speech classifications.

	success	n	p	proportion	95% CI
attack	79	360	< 0.001	0.22	[0.18, 0.27]
defense	320	360	< 0.001	0.89	[0.85, 0.92]

Experiment 2

Multilevel (logistic) models included random intercepts for participants nested within their group to account for violations of independence, since participants made repeated decisions and were part of a group in which they potentially influenced each other's decisions over time. All reported statistical tests were two-tailed.

We fitted a multilevel regression model to examine how one's 'true' position in the conflict (which was common knowledge in the no-deception treatment) impacted followers' contributions to conflict (Table S9). In this model with individual-level contributions to conflict as the dependent variable, we included followers' 'true' position (0 = defender, 1 = attacker) as a fixed factor. This model only included data of participants who were followers and were in the no-deception treatment.

Table S9. Mixed effects regression modeling how one's 'true' position impacted contributions to conflict.

		Estimate	SE	t	p	95% CI
**Model**	intercept	11.67	0.50	23.17	< 0.001	[10.67, 12.67]
	attacker	-3.84	0.20	-19.05	< 0.001	[-4.23, -3.44]

We fitted a multilevel logistic regression model to examine how followers' 'true' position in the conflict impacted free-riding (Table S10). In this model with a dummy variable that coded if followers free-rode (= 1, contributed nothing to conflict) or not (= 0, contributed to conflict) as the dependent variable, we included followers' 'true' position (0 = defender, 1 = attacker) as a fixed factor. This model only included data of participants who were followers and were in the no-deception treatment.

Table S10. Mixed effects logistic regression modeling how one's 'true' position impacted free-riding.

		Estimate	SE	z	p	95% CI
**Model**	intercept	-6.06	0.47	-12.90	< 0.001	[-7.06, -5.19]
	attacker	4.33	0.33	13.00	< 0.001	[3.72, 5.04]

We fitted a multilevel logistic regression model to examine how one's 'true' position in the conflict impacted conflict success (Table S11). In this model with conflict success (0 = no success, 1 = success) as the dependent variable, we included participants' 'true' position (0 = defender, 1 = attacker) as a fixed factor. This model only included data of participants who were in the no-deception treatment.

Table S11. Mixed effects logistic regression modeling how one's 'true' position impacted conflict success.

		Estimate	SE	z	p	95% CI
**Model**	intercept	1.12	0.10	10.99	< 0.001	[0.92, 1.32]
	attacker	-2.24	0.14	-15.55	< 0.001	[-2.52, -1.96]

We fitted a multilevel regression model to examine how participants' 'true' position in the conflict impacted earnings (Table S12). In the model with conflict earnings as the dependent variable, we included participants' 'true' position (0 = defender, 1 = attacker) as a fixed factor. The model only included data of participants who were in the no-deception treatment and who were leaders.

Table S12. Mixed effects regression modeling how leaders' 'true' position impacted their earnings.

		Estimate	SE	t	p	95% CI
**Model**	intercept	30.15	0.92	32.93	< 0.001	[28.36, 31.95]
	attacker	-20.31	1.04	-19.49	< 0.001	[-22.35, -18.26]

We fitted a multilevel regression model to examine how treatment impacted followers' contributions to conflict (Table S13). In the model with individual-level contributions to conflict as the dependent variable, we included treatment (0 = no-deception, 1 = deception), round, and their interaction as fixed factors. This model only included data of participants who were followers.

Table S13. Mixed effects regression modeling how treatment impacted contributions to conflict over time.

		Estimate	SE	t	p	95% CI
**Model**	intercept	11.29	0.51	22.15	< 0.001	[10.30, 12.29]
	deception	-0.06	0.72	-0.09	0.929	[-1.47, 1.34]
	round	-0.15	0.02	-8.63	< 0.001	[-0.18, -0.11]
	deception × round	0.20	0.02	8.31	< 0.001	[0.15, 0.25]

We fitted a multilevel logistic regression model to examine how one's 'true' position in the conflict impacted leader deception (Table S14). In this model, with a dummy variable coding if leaders communicated their group's position dishonestly (= 1) or honestly (= 0) as the dependent variable, we included leaders' 'true' position (0 = defender, 1 = attacker) as a fixed factor. This model only included data of participants who were leaders and were in the deception treatment.

Table S14. Mixed effects logistic regression modeling how leaders' 'true' position impacted deception.

		Estimate	SE	z	p	95% CI
**Model**	intercept	-2.37	0.26	-9.17	< 0.001	[-2.93, -1.87]
	attacker	1.75	0.18	9.83	< 0.001	[1.40, 2.11]

We fitted a multilevel regression model to examine how attacking leader (dis)honesty impacted their expectations about their followers' contributions to conflict (Table S15). In this model, with leaders' expectations about their followers' contributions to conflict as the dependent variable, we included a dummy variable coding if attacking leaders communicated their group's position dishonestly (= 1) or honestly (= 0) as a fixed factor. This model only included data of participants who were leaders, whose 'true' position was attacker, and who were in the deception treatment.

Table S15. Mixed effects regression modeling how attacking leader (dis)honesty impacted their expectations about followers' contributions to conflict.

		Estimate	SE	t	p	95% CI
**Model**	intercept	10.95	0.40	27.15	< 0.001	[10.16, 11.74]
	dishonest	1.89	0.36	5.31	< 0.001	[1.19, 2.58]

We fitted a multilevel regression model to examine how attacking leader (dis)honesty impacted followers' actual contributions to conflict (Table S16). In this model, with individual-level contributions to conflict as the dependent variable, we included a dummy variable coding if attacking leaders communicated their group's position dishonestly (= 1) or honestly (= 0) as a fixed factor. This model only included data of participants who were followers, whose 'true' position was attacker, and who were in the deception treatment.

Table S16. Mixed effects regression modeling how attacking leader (dis)honesty impacted contributions to conflict.

		Estimate	SE	t	p	95% CI
**Model**	intercept	9.72	0.55	17.53	< 0.001	[8.62, 10.82]
	dishonest	3.57	0.31	11.62	< 0.001	[2.96, 4.17]

We fitted a multilevel logistic regression model to examine how attacking leader (dis)honesty impacted follower free-riding (Table S17). In this model with a dummy variable that coded if followers free-rode (= 1; contributed nothing to conflict) or not (= 0; contributed to conflict) as the dependent variable, we included a dummy variable coding if attacking leaders communicated their group's position dishonestly (= 1) or honestly (= 0) as a fixed factor. This model only included data of participants who were followers, whose 'true' position was attacker, and who were in the deception treatment.

Table S17. Mixed effects logistic regression modeling how attacking leader (dis)honesty impacted free-riding.

		Estimate	SE	z	p	95% CI
**Model**	intercept	-3.06	0.44	-6.93	< 0.001	[-5.30, -2.44]
	dishonest	-3.03	0.47	-6.49	< 0.001	[-4.86, -2.10]

We fitted a multilevel logistic regression model to examine how attacking leader (dis)honesty impacted conflict success (Table S18). In this model with conflict success (0 = no success, 1 = success) as the dependent variable, we included a dummy variable coding if attacking leaders communicated their group's position dishonestly (= 1) or honestly (= 0) as a fixed factor. This model only included data of participants whose 'true' position was attacker and who were in the deception treatment.

Table S18. Mixed effects logistic regression modeling how attacking leader (dis)honesty impacted conflict success.

		Estimate	SE	z	p	95% CI
**Model**	intercept	-1.25	0.11	-11.47	< 0.001	[-1.46, -1.04]
	dishonest	0.88	0.17	5.17	< 0.001	[0.55, 1.22]

We fitted a multilevel logistic regression model to examine how treatment impacted attackers' conflict success (Table S19). In this model with conflict success (0 = no success, 1 = success) as the dependent variable, we included treatment (0 = no-deception, 1 = deception) as a fixed factor. This model only included data of participants whose 'true' position was attacker.

Table S19. Mixed effects logistic regression modeling how treatment impacted attacker conflict success.

		Estimate	SE	z	p	95% CI
**Model**	intercept	-1.15	0.12	-9.47	< 0.001	[-1.40, -0.91]
	deception	0.57	0.16	3.52	< 0.001	[0.25, 0.91]

We fitted a multilevel regression model to examine how attacking leader (dis)honesty impacted earnings (Table S20). In this model with conflict earnings as the dependent variable, we included a dummy variable coding if attacking leaders communicated their group's position dishonestly (= 1) or honestly (= 0) as a fixed factor. This model only included data of participants whose 'true' position was attacker, who were in the deception treatment, and who were followers.

Table S20. Mixed effects regression modeling how attacking leader (dis)honesty impacted followers' earnings.

		Estimate	SE	t	p	95% CI
**Model**	intercept	13.05	0.65	19.95	< 0.001	[11.75, 14.35]
	dishonest	-1.88	0.40	-4.69	< 0.001	[-2.67, -1.09]

We fitted a multilevel regression model to examine how treatment impacted attackers' earnings (Table S21). In this model with conflict earnings as the dependent variable, we included treatment (0 = no-deception, 1 = deception) as a fixed factor. This model only included data of participants whose 'true' position was attacker and who were leaders.

Table S21. Mixed effects regression modeling how treatment impacted attacking leaders' earnings.

		Estimate	SE	t	p	95% CI
**Model**	intercept	9.85	1.06	9.26	< 0.001	[7.76, 11.93]
	deception	4.69	1.50	3.12	0.002	[1.75, 7.64]

We fitted a multilevel regression model to examine how attacking leader (dis)honesty impacted earnings (Table S22). In this model with conflict earnings as the dependent variable, we included a dummy variable coding if attacking leaders communicated their group's position dishonestly (= 1) or honestly (= 0) as a fixed factor. This model only included data of participants whose 'true' position was attacker, who were in the deception treatment, and who were leaders.

Table S22. Mixed effects regression modeling how attacking leader (dis)honesty impacted leaders' earnings.

		Estimate	SE	t	p	95% CI
**Model**	intercept	12.04	1.29	9.30	< 0.001	[9.47, 14.57]
	dishonest	6.44	1.78	3.61	< 0.001	[2.87, 9.95]

We fitted a multilevel regression model to examine how leader (dis)honesty and follower earnings impacted how many points followers donated to their leader (Table S23). In this model with points donated to the leader as the dependent variable, we included the total number of rounds that the leader was honest and participants' total earnings as fixed factors. The model only included data of participants who were followers and who were in the deception treatment.

Table S23. Mixed effects regression modeling how leader (dis)honesty and follower earnings impacted donations to their leader.

		**Estimate**	**SE**	**t**	**p**	**95% CI**
**Model**	intercept	5.58	1.11	5.04	< 0.001	[3.52, 7.68]
	total honesty	-0.05	0.06	-0.77	0.444	[-0.19, 0.08]
	total earnings	0.02	0.004	3.58	0.001	[0.01, 0.03]

Methods S4

Deviations from pre-registration

We deviated from the pre-registration of Experiment 1 with regard to (i) the political experts and (ii) the exclusion criteria.

Political experts

We emailed 91 academic colleagues with expertise in political science, conflict studies, and international relations. Our email briefly introduced ourselves and the study aims, and asked each expert to participate in a short, 20-minute online survey. The link to the survey was embedded in the email. Our initial invite in January 2023 resulted in nine participations. A reminder one month later resulted in another five participants. The low response rate raised concerns about possible self-selection and prohibited performing the planned analyses for pre-registered hypotheses 3 and 4.

Figure S1 shows the classification of speech excerpts as either revisionist attack or non-revisionist defense by political experts (n = 14). Political experts classified attacker (70.2%) and defender (59.5%) speeches correctly in the majority of cases, while they only misclassified 29.8% of attacker and 40.5% of defender speeches. In addition, political experts did not differ in their confidence (on a scale from 1-7) in their classification between attack and defense, regardless of the actual (MLM, b = 0.0004, p = .998, 95% CI [-0.43, 0.43]) or predicted position (MLM, b = -0.12, p = .595, 95% CI [-0.57, 0.32]) of the speech excerpt.

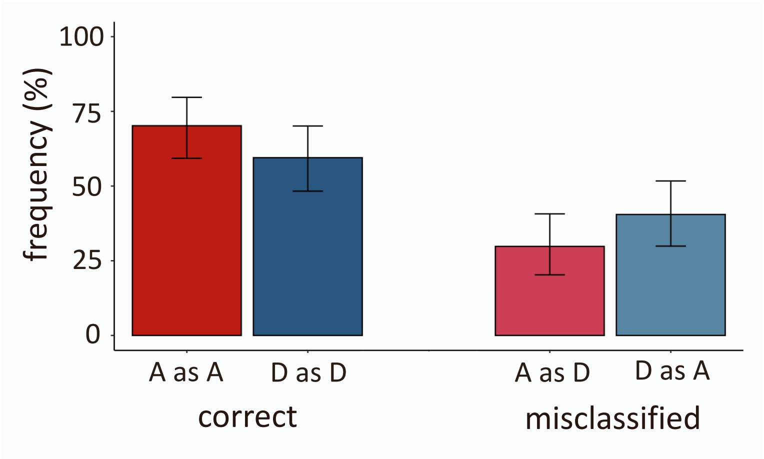

Figure S1. Experts are better at classifying attack and defense speech excerpts. Classification of attacker and defender speeches. Experts (n = 14) correctly classified the majority of attacker (70.2%) and defender (59.5%) speeches, while they only misclassified 29.8% of attacker and 40.5% of defender speeches. Error bars indicate the standard error of the mean.

Exclusion criteria

According to our pre-registration, we planned to exclude participants who recognized two or more speech excerpts from the analyses. After applying this criterion, only 164 (out of the original 252) participants remained. Since the results were consistent both with and without these participants, we decided to include all participants in the main analysis to maximize statistical power. Below, we provide the results for excluding participants who recognized two or more speech excerpts (n = 164).

We fitted a multilevel regression model to examine how the predicted and 'true' position of a leader impacted participants' support for this leader's causes (Table S24). In this model with leader support (on a scale from 1-7) as the dependent variable, we included the predicted position (0 = revisionist attacker, 1 = non-revisionist defender) and 'true' position (0 = revisionist attacker, 1 = non-revisionist defender) of the leader as fixed factors.

Table S24. Mixed effects regression modeling how the predicted and 'true' position of a leader impacted support for their causes.

		**Estimate**	**SE**	**t**	**p**	**95% CI**
**Model**	intercept	2.69	0.10	27.77	< 0.001	[2.50, 2.88]
	predicted defender	1.86	0.06	31.15	< 0.001	[1.74, 1.98]
	actual defender	0.04	0.06	0.77	0.444	[-0.07, 0.15]

We fitted two multilevel binomial regression models to assess how accurately participants classified leader speeches as either attack or defense motivated (Table S25). Specifically, we tested whether the proportion of correct classifications deviated significantly from chance (0.5). In the first model, we examined the correct classification of attacker speeches. In the second model, we examined the correct classification of defender speeches.

Table S25. Multilevel binomial tests for classifying attacker and defender speeches.

		Estimate	SE	z	p	95% CI
**Model**	attack	-0.15	0.07	-2.07	0.039	[-0.30, -0.01]
	defense	0.92	0.09	10.71	< 0.001	[0.76, 1.10]
